# Epilepsy and Bilingualism. A Systematic Review

**DOI:** 10.3389/fneur.2019.01235

**Published:** 2019-11-26

**Authors:** Lisa Bartha-Doering, Silvia Bonelli

**Affiliations:** ^1^Department of Pediatrics and Adolescent Medicine, Medical University of Vienna, Vienna, Austria; ^2^Comprehensive Center for Pediatrics, Medical University of Vienna, Vienna, Austria; ^3^Department of Neurology, Medical University of Vienna, Vienna, Austria

**Keywords:** language localization, epilepsy, bilingualism, functional imaging, seizures, language proficiency, second language acquisition

## Abstract

**Background:** In patients with epilepsy, language abilities and neural language organization have been primarily investigated for the patient's mother tongue. However, in clinical practice, many patients use more than one language or use their second language more than their mother tongue. Yet, information about the linguistic profiles and brain organization of both languages in bilingual epilepsy patients is scarce. The purpose of this study was thus to systematically review the literature on language localization and language abilities in bilingual patients with epilepsy.

**Methods:** An extensive literature search was performed using various electronic databases, including Embase and Medline. Key aspects of inclusion criteria were the assessment of language abilities and/or the investigation of neural language mapping in bilingual patients with epilepsy.

**Results:** Our search strategy yielded 155 articles on language in bilingual epilepsy patients. Of these, 12 met final eligibility criteria. The majority of included articles focused on brain mapping of language using fMRI, Wada-test, or electrocortical stimulation in bilingual epilepsy patients, five studies investigated interictal language abilities in this patient group. Study results showed a pronounced heterogeneity of language abilities in bilingual patients, varying from intact language profiles to impairment in several language functions in both languages. However, the mother tongue was most often better perserved than the second language. Furthermore, studies on brain mapping of both languages again revealed heterogeneous findings ranging from identical brain regions for both languages to overlapping, but more distributed cortical areas for the non-native language.

**Conclusions:** This review underlines the need to evaluate linguistic abilities in both languages, as well as the necessity to preoperatively map both languages in bilingual epilepsy patients. In contrast to the large scientific interest in language abilities and language localization in monolingual epilepsy patients, this review shows that in bilingual patients, the examination of language functions and the identification of brain regions associated with both languages so far played a minor role in epilepsy research. Our review thus emphasizes the need of future research activities in this field.

## Introduction

In the context of globalization and immigration, more and more people are exposed to languages other than their mother tongue. In a survey conducted by the European Commission in 2012, 54 percent of participants are able to hold a conversation in at least one additional language, with increasing rates (1). All over the world, two thirds of children grow up in a bilingual environment (2).

Bilingualism encompasses a heterogeneous typology of speakers. The acquisition of two (or more) languages may occur in different contexts, at different ages, in different situations, with different stimuli and learning environments, and at different proficiency levels. For the present review, we use a broad definition of bilingualism: We define a bilingual person as somebody who can communicate efficiently in both languages. This person may not have an equal proficiency of different language modalities in both languages and may not have a perfect knowledge of their respective cultures, but may be able to express themselves efficiently in two languages.

Several studies in healthy adults suggest that bilingualism is associated with structural brain modulations. Gray matter volume and density studies found significant gray matter increases in bilinguals compared to monolinguals in left inferior temporal and left parietal regions (3, 4), the left anterior cingulate cortex (5), and the cerebellum (6). Increased cortical thickness for bilinguals as compared to monolinguals was observed in the left inferior frontal gyrus (7). In addition, modulations in white matter regions were described: however, whereas some studies found increased fractional anisotropy in parts of the corpus callosum and the inferior fronto-occipital fasciculus in bilinguals (8, 9), others reported decreased fractional anisotropy in these two regions (10, 11). The degree of structural brain alterations in bilinguals has shown to be proportional to second language experience (12).

Functional imaging studies for brain mapping of both languages in healthy bilinguals show controversial findings. Some studies evidenced that second language processing used the first language's functional brain networks located predominantly in inferior frontal, middle and superior temporal, and parietal areas of the left hemisphere (13–15). Activations in these areas have often found to be higher in bilinguals as compared to monolinguals, which has been explained with higher processing demands to monitor both languages (16–18). On the contrary, other studies proved that processing of a second language involved additional functional brain areas in bilinguals (19–22). These additional functional brain areas were predominantly located in homologous areas of the right hemisphere, resulting in a weaker language lateralization in bilinguals as compared to monolinguals. Two studies furthermore examined both, structural and functional relationships between gray matter regions in bilingual healthy adults and pointed to the important role of the left inferior frontal gyrus and its stronger functional connections to temporo-parietal brain regions in bilinguals as compared to monolinguals (9, 23).

In healthy individuals, several factors have been identified that may play a role in determining whether both language networks overlap or differ, among them the age of acquisition (24, 25), second language learning strategies (26), the level of proficiency (27), and the orthographic transparency of the second language, i.e., the systematicity in the mapping between graphemes and phonemes (25).

Many epilepsy patients exhibit language deficits, with naming and spontaneous speech being most often affected (28). These deficits may increase with longer duration of epilepsy (29). Epilepsy surgery is widely accepted as an effective therapeutic alternative in patients with medically refractory epilepsy. Surgical therapy has shown to result in favorable outcomes, concerning seizure activity as well as cognitive aspects. Hereby, knowledge about preoperative language abilities and preoperative language localization plays a major role. However, despite the global predominance of multilingualism, much remains unknown regarding functioning and brain mapping of both languages in bilingual epilepsy patients. Most studies so far have concentrated on language abilities and language mapping in monolingual epilepsy patients, or have neglected the fact that their patients used a second language besides their mother tongue. Nevertheless, for an optimal outcome, presurgical brain mapping has to take into account both languages. We therefore aimed to conduct a systematic review of studies investigating abilities and brain mapping of both languages in bilingual patients with epilepsy to offer the current state of research and potentially initiate further research activities in the field of language assessment in bilingual epilepsy.

## Methods

We conducted a comprehensive search for empirical studies that investigated language localization or language abilities in bilingual epilepsy patients. Publication year and language were not restricted. Studies were identified by searching the following electronic databases up to the 17th of January 2019: Arts and Humanities Citation Index, Biosis Previews, Cochrane Central Register of Controlled Trials, Cochrane Database of Systematic Reviews, Conference Proceedings Citation Index—Science, Conference Proceedings Citation Index—Social Science and Humanities, Current Contents Connect, EMBASE, ERIC, MEDLINE, PsycINFO, PSYNDEXPlus, Science Citation Index Expanded, and Social Sciences Citation Index. The following search terms were used: (bilingual^*^ OR second language^*^ OR two language^*^ OR dual language^*^ OR (L2 AND (language^*^ OR proficien^*^ OR learn^*^))) AND (epilep^*^ OR seizure^*^).

Articles were included if (a) they provided original data on interictal language abilities and/or language mapping in bilingual epilepsy patients, and (b) studies described quantitative results in form of counts or numbers (ref chapter Data Extraction). Eligibility assessment was performed independently in an unblinded standardized manner by both authors. Disagreements between reviewers were resolved by consensus.

### Data Extraction

One reviewer (LBD) extracted information from the included papers, the second author checked the extracted data. Disagreements were resolved by discussion between the two review authors. Data items compromised (1) characteristics of epilepsy patients (including age, education, seizure lateralization, age at epilepsy onset, duration of epilepsy, MRI findings, drug resistence); (2) characteristics about their languages (L1, L2, age at first exposure to L2, duration of exposure to L2, L2 proficiency); (3) information about controls; (4) languages used during testing; (5) interictal language abilities tested; (6) interictal language tests used; (7) methods used to map language functions; (7) language mapping test paradigm; and (8) results.

Due to the large variation in methodology and the limited amount of data, a quantitative meta-analysis of study results was not feasible. We therefore analyzed these data qualitatively. The PRISMA guidelines were used as a framework for this review (30).

## Results

**Figure 1:**
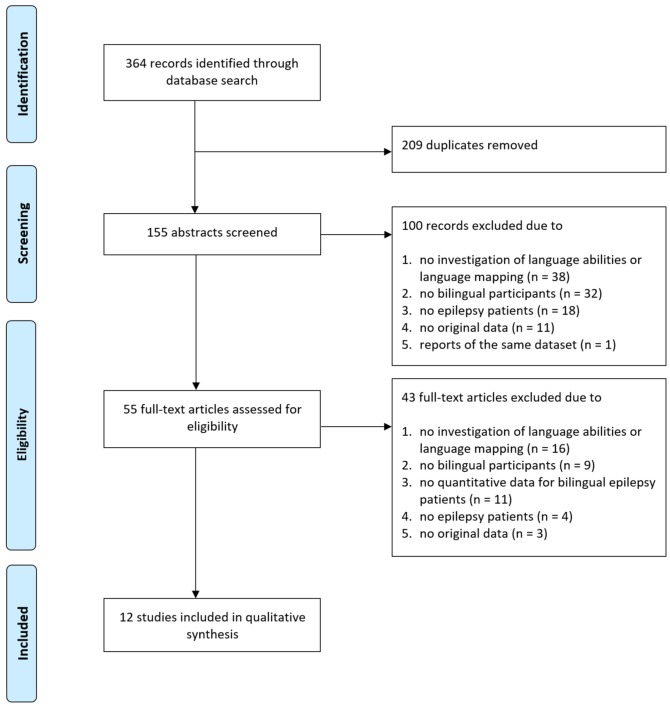
Flow diagram depicting search process and study selection.

Literature search yielded, after elimination of duplicates, 155 articles (Figure 1). After screening of all abstracts, 100 records were excluded. Thus, 55 articles were included in the full-text analysis. Of these, 43 full-text articles were excluded (Figure 1 depicts reasons for exclusion per screening step). Overall, articles were excluded due to the following exclusion criteria^1^: no investigation of interictal language abilities or language mapping described (*n* = 54), no bilingual participants included (*n* = 41), no epilepsy patients included (*n* = 22), no original data reported (*n* = 14), no quantitative data for bilingual epilepsy patients presented (*n* = 11), or reporters of the same dataset (*n* = 1). Finally, 12 studies were identified meeting inclusion and not meeting exclusion criteria.

### Study Participants

#### Epilepsy Patients

Overall, 129 bilingual epilepsy patients were investigated, including six participants younger than 18. Sample size varied between studies from 1 to 56 epilepsy patients with ages from 13 to 53 years (overall mean age 30.52, *sd* 9.48; Table 1). Seizures were left lateralized in 79 patients and could be further localized to the temporal lobe in 24, to the frontal lobe in two, and to the occipital lobe in one of them. Seizures were right lateralized in 28 epilepsy patients, with 12 of them originating from the right temporal lobe and one from the frontal lobe. In addition, seven participants experienced bilateral seizure activity, six patients suffered from generalized seizures, and nine bilingual epilepsy patients had an unknown seizure lateralization. The majority of patients in the studies that provided information on clinical and demographic variables had a mean epilepsy duration of more than 10 years and resistance to antiepileptic drugs in their population (overall mean age at epilepsy onset 15.11 years, *sd* 9.86, range 0.4–47; overall mean duration of epilepsy 15.28 years, *sd* 12.45, range 1–50). MRI findings were heterogeneous. In sum, 36% of patients across all studies with MRI examinations displayed mesiotemporal/hippocampal sclerosis, 22% suffered from tumors, 40% had other structural findings including dysplasia and cavernoma, and 2% presented with a normal MRI scan.

**Table 1:** Study characteristics.

**References**	**Sample**	**Seizure lateralization (patients, *n*)**	**Age, y**	**Education**	**Age at epilepsy onset, y**	**Duration of epilepsy, y**	**MRI findings (patients, *n*)**	**Drug resistance (*n*)**	**L1 (patients, n)^**a**^**	**L2 (patients, n)**	**Age at L2 first exposure, y**	**Duration of exposure to L2, y**	**L2 proficiency (patients, *n*)**
Gooding et al. (31)	56	L (23) R (14) BL (5) GEN (6) UNKN (8)	36.9 (14.3)	15.7 y (2.5)	22.7 (1.8)	Not spec	Not spec	Not spec	Spanish (22) Creole (3) Italian (3) Korean (3) Yiddish (3) French (2) Greek (2) Telugu (2) other (16)^**a**^	English (56)	Not spec	Not spec	Not spec
O'Grady et al. (32)	1	R	33	5 y	5	28	Normal	Not spec	Urdu	English	8	25	Not spec
Tomasino et al. (33)	1	L	30	17 y	25	5	Glioma grade II	na	Serbian	Italian	28	2	High^b^
Centeno et al. (34)	16	LT (5) RT (3) T (2) LF (2) RF (1) LOC (1) L (1) UNKN (1)	34.3 (7.8)	Not spec	13.8 (9.8)	21.0 (15.3)	HS (6) Cryptogenic (4) Cavernoma (3) FCD (1) Unclear (1) Dual pathology (1)	16	Portuguese (3) Urdu (2) Polish (2) Turkish (2) Other (7)^**a**^	English (16)	Before 6 (5) After 6 (11)	Not spec	Low (5)^c^ Medium (6) High (5)
Cervenka et al. (35)	4	LT (4)	39 (11.1)	Not spec	22.2 (10.0)	16.8 (16.3)	Gangliocytoma (1) MTS (1)	4	Igbo (1) Italian (1) Spanish (1) Greek (1)	English (4)	12.0 (4.9)	27.0 (11.1)	Not spec
Wang et al. (36)	1	L	25	Graduate student	Not spec	Not spec	Glioma	Not spec	Chinese	English	13	12	High
Serafini et al. (37)	1	LT	13	Student	11	2	Astrocytoma	1	English	Hebrew	Infancy	Not spec	Raised bilingual since infancy
Navarro et al. (38)	1	RT	34	At least 12, not further spec	8	26	HS	1	French	English	11	8	Low
Cheung et al. (39)	21	LT (13) RT (8)	26.3 (9.1)	LT: 11.3 y (2.7) RT: 10.6 y (3.0)	15.0 (9.2)	11.3 (7.9)	MTS (8) Glioma (4) Cyst (3) Hemangioma (2) DNET (1) Astrocytoma (1) Lesion (1)	21	Chinese (21)	English (21)	Before 6	At least 10	Not spec
Lucas et al. (40)	25	L (24) R (1)	31.0 (8.9)	Not spec	Not spec	Not spec	Not spec	Not spec	English (14) Spanish (3) Other (5)^a^ Missing (3)	English (8) Spanish (6) Korean (2) Other (6)^a^ Missing (3)	Not spec	Not spec	>65%^d^
Trudeau et al. (41)	1	L	17	Not spec	5	12	Rasmussen encephalitis	1	English	French	0	17	Raised bilingual from birth on
Pouratian et al. (42)	1	LT	43	Not spec	39	4	Low grade tumor	1	Spanish	English	6	37	Language at home and work

BL, bilateral; DNET, dysembryoplastic neuroepithelial tumor; FCD, focal cortical dysplasia; GEN, generalized; L1, mother tongue; L2, second language; L, left hemisphere; LF, left frontal; LOC, left occipital; LT, left temporal; HS, hippocampal sclerosis; MTS, mesial temporal sclerosis; na, not applicable; Not spec, not specified; R, right hemisphere; RF, right frontal; RT, right temporal; SD, standard deviation; T, temporal; UNKN, unknown.

a Languages with just 1 speaker are presented as “other.”

a As administered by the Bilingual Language History Questionnaire (43).

c Self-rated.

d *Proficiency score*.

#### Control Groups

The majority of studies did not compare findings in bilingual epilepsy patients to a control group, only three studies investigated group differences. One of them compared bilingual epilepsy patients to monolingual epilepsy patients (31), one compared bilingual epilepsy patients to bilingual healthy controls (39), and one compared a bilingual epilepsy patient with monolingual healthy controls (33). All studies that investigated language abilities in their patients interpreted their findings in relation to normative test control data, though not for all language abilities tested.

### Information About the Patients' Languages

#### First Language (L1)

In 78 epilepsy patients, L1 was an Indo-European language. Within these, Iberian languages (Spanish, Portuguese) were the ones most often learned as first language (*n* = 32). Further languages within the Indo-European language family comprised Germanic (English, Dutch, Yiddish), Romance (Italian, French, Romanian), Hellenic (Greek), Italic (Welsh), Balto-Slavic (Polish, Serbian, Russian), and Indo-Iranian languages (Urdu, Hindi, Gujarati, Bengali, Farsi). In 26 participants, L1 belonged to the Sino-Tibetian language family (Chinese, Mandarin, Cantonese), and four patients spoke Korean as L1. Further L1 belonged to the Tai Kadai (Lao), Turkic (Turkish), Niger-Congo (Shona, Igbo), Japonic (Japanese), Dravidian (Telugo, Malayan, Tamil), and Afro-Asiatic (Arabic, Eritrea) language families, and not further specified Creol languages.

#### Second Language (L2)

Most often, L2 of study participants was English (*n* = 109). Further language families of L2 within the Indo-European languages comprised Romance (Italian, French), Iberian (Spanish), Hellenic (Greek), Uralic (Finnish), and other Germanic languages (German), besides English. There were only few study participants with their L2 belonging to a language family other than Indo-European (*n* = 5), including Korean, Sino-Tibetian (Cantonese), Aftro-Asiatic (Hebrew), and Austro-Asiatic (Vietnamese) language families.

Ten studies specified the age of the first exposure to L2 in overall 48 participants. Twenty-eight of them acquired their L2 before the age of six, 20 patients were first exposed to L2 with 6 years of age or later. Years of exposure to L2 was described in eight studies and in overall 31 participants, with 29 of them having more than 10 years of L2 exposure. Eight studies furthermore informed about L2 proficiency and described low and medium proficiency, respectively, in six participants each, and high proficiency in 35 study participants (75%).

Overall, study participants most often spoke Indo-European languages. However, the whole study sample in this review comprises a wide variety of languages, especially for L1. In about 1/3 of study participants, more detailed information was available about age at first exposure to L2 and years of exposure to L2. Most of them had more than 10 years of exposure to L2 and spoke this second language with high proficiency.

### Interictal Language Abilities

Five studies investigated the interictal language abilities of bilingual epilepsy patients, and overall, 63 patients were tested with language tests tapping different language functions (31–33, 35, 41); Table 2. In four studies, language abilities were tested in both L1 and L2, one study examined linguistic functions in L2 only (31). One study (41) reported post-operative language abilities in a single patient, whereas the other four studies investigated language abilities in non-operated patients.

**Table 2:** Interictal language abilities and/or language localization in bilingual epilepsy patients.

**References**	***N***	**Controls**	**Test language**	**Interictal language abilities tested**	**Interictal language tests used**	**Language localization methods**	**Language localization test paradigm**	**Results**
Gooding et al. (31)	56	186 ML E	L2	Visual naming, auditory naming, phonemic fluency, semantic fluency, word reading	AVNT BNT WTAR COWAT	–	–	BLING epilepsy patients scored significantly worse in L2 (English) naming compared to native English speaking ML epilepsy patients. No differences between groups were found in other language abilities. An association between seizure laterality and naming abilities was only significant within the ML group
O'Grady et al. (32)	1	0	L1 L2	Comprehension, visual naming, semantic fluency	NAB PPVT	fMRI, dichotic listening	Sentence reading and comprehension, letter fluency, antonym generation, object naming, word perception (Dichotic Listening)	This patient with right hemisphere epilepsy showed reduced language abilities in both L1 and L2. FMRI revealed left lateralized, but bilateral activations in frontal, temporal, and parietal areas for both languages. Dichotic listening showed a left ear advantage for receptive language processing. These findings point to a right hemisphere involvement for both languages
Tomasino et al. (33)	1	18 ML HC	L1 L2	Comprehension, phonemic discrimination, visual naming, word and pseudoword reading, word and pseudoword repetition	Token Test BADA	Electrocortical intraoperative stimulation, fMRI	Counting, object naming, silent object naming, verb generation	The patient had intact language abilities in both L1 and L2, only naming was worse in L2 compared to L1. Electrocortical intraoperative stimulation in the left superior temporal gyrus induced involuntary language switching from L2 to L1, stimulation in inferior frontal gyrus induced speech arrest. In fMRI, L1 and L2 both activated the left superior temporal gyrus and the left supramarginal gyrus. Thus, this epilepsy patient showed overlapping language areas for L1 and L2
Centeno et al. (34)	16	0	L1 L2	–	–	fMRI	Verbal fluency, verb generation	At the group level, L2 revealed overlapping language areas with L1, but larger clusters and a more bilateral distribution. At the individual level, language laterality indices were concordant between L1 and L2 except in one participant
Cervenka et al. (35)	4	0	L1 L2	Naming, spontaneous speech, writing, reading, comprehension	BNT WRAT Token Test	Subdural electrocortical stimulation, electrocorticography	Object naming	L1 and L2 language assessment revealed borderline to average language abilities in all patients, no language impairment. Electrocortical mapping during naming in L1 and L2 revealed both shared and distinct areas in three patients. More language sites in L2 than in L1 were found in two patients
Wang et al. (36)	1	0	L1 L2	–	–	Electrocortical intraoperative stimulation	Object naming, naming of colors or shapes	Stimulation of the left caudate induced difficulties in language switching
Serafini et al. (37)	1	0	L1 L2	–	–	Subdural electrocortical stimulation	Object naming, sentence completition, reading	This patient showed distinct but also overlapping cortical areas for L1 and L2
Navarro et al. (38)	1	-	L1* L2	–	–	fMRI	auditory semantic decision	FMRI in L2 activated a bihemispheric, but right lateralized language network in frontal, temporal, and parietal regions, including the right hippocampus. Seizures affecting the right hippocampus elicited L2 ictal speech automatisms
Cheung et al. (39)	21	23 BLING E HC	L1 L2	–	–	fMRI	Reading words	RTLE and HC showed left lateralized activations in reading English words (L2) and bilateral activations in reading Chinese characters (L1). LTLE revealed bi-hemispheric involvement during reading in both languages
Lucas et al. (40)	25	–	L1 L2	–	–	Electrocortical intraoperative stimulation	Object naming	Intraoperative cortical stimulation in the dominant hemisphere revealed distinct language-specific sites, but also shared language sites. L2-specific sites were located exclusively in the posterior temporal and parietal lobes, whereas shared sites and L1-specific sites were located throughout the mapped cortical areas
Trudeau et al. (41)	1	–	L1 L2	Comprehension, repetition, naming, fluency, reading, writing	BDAE Token Test TLDD EVIP-A	–	–	After left hemispherectomy, the patient showed severe language deficits in most language abilities in both L1 and L2. However, linguistic profiles of L1 and L2 were not identical
Pouratian et al. (42)	1	–	L1 L2	–	–	fMRI, electrocortical intraoperative stimulation, intraoperative optical imaging	Object naming	Cortical language representations of L1 and L2 consisted of both overlapping and distinct language areas

*Results were only reported for L2.

*AVNT, Auditory and Visual Naming Tests; BADA, Battery for the Analysis of Aphasic Deficits; BDAE, Boston Diagnostic Aphasia Examination; BLING, bilingual; BNT, Boston Naming Test; COWAT, Controlled Oral Word Association Test; EVIP-A, Échelle de Vocabulaire en Image Peabody; HC, healthy controls; L1, mother tongue; L2, second language; LTLE, left temporal lobe epilepsy patients; ML, monolingual; E, epilepsy patients; fMRI, functional magnetic resonance imaging; NAB, Neuropsychological Assessment Batteries; PPVT, Peabody Picture Vocabulary Test; RTLE, right temporal lobe epilepsy patients; TLDD, Tests de Langage Dudley-Delage; WTAR, Wechsler Test of Adult Reading; WRAT, Wide Range Achievement Test*.

Most often, visual naming was investigated. Studies found naming in L1 better than in L2 in most, but not all patients (33, 35, 41). Furthermore, visual naming in L2 was significantly worse in bilingual epilepsy patients compared to monolingual epilepsy patients (31). Overall, 31% of bilingual epilepsy patients across all studies revealed impaired naming performance in L1, 84% of all patients exhibited impaired naming performance in L2.

Verbal fluency was investigated in four studies and in overall 62 patients. Cervenka et al. (35) found L2 verbal fluency below the 5th percentile in one out of four patients, and Gooding et al. (31) described the group performances of phonemic and semantic fluency in L2 in bilingual epilepsy patients not significantly different to monolingual epilepsy patients. Verbal fluency in L1 was only investigated in two single-case studies and reported to be at borderline in one patient (32) and severely impaired in the second patient who suffered from Rasmussen encephalitis and had a hemispherectomy (41).

Four studies investigated reading abilities of their bilingual patients and investigated overall 62 patients (31, 32, 35, 41). Only one study reported impaired reading abilities in both L1 and L2 in their patient with Rasmussen encephalitis and hemispherectomy (41), the other studies found intact reading abilities in both languages (33, 35) and no differences between L2 reading in bilingual epilepsy patients compared to monolingual epilepsy patients (31).

Only two studies investigated writing abilities of their patients. The single case with Rasmussen encephalitis and hemispherectomy showed severe writing deficits for both languages, whereas group comparisons between L2 writing in bilingual vs. L1 writing in monolingual epilepsy patients did not yield significant differences (31).

Auditory comprehension for L1 was investigated in two bilingual epilepsy patients, and comprehension for L2 was examined in five bilingual epilepsy patients only. Tomasino et al. (33) found both L1 and L2 comprehension intact in their case of bilingual epilepsy, whereas Trudeau et al. (41) described auditory comprehension impaired for both languages in their patient after hemispherectomy. Overall, comprehension of L2 was impaired in 3/5 epilepsy patients (32, 33, 35, 41).

In sum, in non-operated epilepsy patients, linguistic abilities in L1 were often better preserved than in L2, however, only few studies investigated the interictal language abilities of bilingual epilepsy patients, and heterogeneous findings were presented.

### Brain Mapping of Languages

Ten studies performed language mapping in bilingual epilepsy patients, three of them with multiple methods. Language regions were investigated for both languages in all studies (though one study only reported results of L2 mapping) and in overall 71 bilingual epilepsy patients. Six studies used functional magnetic resonance imaging (fMRI) (32–34, 38, 39, 42), four studies performed language mapping with intraoperative electrocortical stimulation during awake surgery (33, 36, 40, 42), two studies investigated languages sites using subdural electrocortical stimulation extraoperatively (35, 37), one study used electrocorticography to detect task-specific spectral perturbations (35), one study used intraoperative optical imaging (42), and one study measured language lateralization with a Dichotic Listening Test (32).

The fMRI paradigms used in the included studies were heterogeneous. Tasks of reading, comprehension, fluency, generation of verbs or antonyms, naming, and auditory semantic decision were requested during scanning. During intraoperative electrocortical stimulation, object naming was examined in all studies. Tomasino et al. (33) tested counting in addition, Wang et al. (36) investigated naming of colors and shades in addition to object naming intraoperatively. Object naming was also investigated in all three studies that used extraoperative subdural stimulation. Serafini et al. (37) furthermore tested sentence completion and reading. Cervenka et al. (35) measured intraoperative electrocorticography and Pouratian et al. (42) used intraoperative optical imaging during naming in addition to intraoperative cortical stimulation. O'Grady et al. (32) examined ear advantages during word perception besides several fMRI paradigms.

Overlapping cortical areas for L1 and L2 were described in two patients (32, 33). In further two single cases (35) and a group study of 16 patients (34), a larger and more bihemispheric distribution for L2 was reported. The latter study, however, found concordant lateralization indices between the two languages in 15/16 study participants. Four single cases (35, 37, 42) and a group of 25 patients (40) exhibited some shared, but also distinct language areas for L1 and L2. Within this group of 25 study participants, L2-specific sites were exclusively located in posterior temporal and parietal regions, whereas shared language sites and L1-specific cortical areas were found to be more distributed.

#### Influence of Seizure Lateralization on Language Lateralization

Most of the reviewed studies did not analyze their data according to seizure lateralization. However, Cheung et al. (39) reported the impact of seizure laterality on language lateralization: Whereas eight right temporal lobe epilepsy patients showed bilateral language representations for L1 during reading of Chinese characters and left language lateralization for L2 during English reading, 13 left temporal lobe epilepsy patients exhibited bilateral language areas for both L1 and L2.

#### Influence of Age of L2 Acquisition on Language Regions in the Brain

Centeno et al. (34) showed that late L2 acquisition (after 6 years of age) was associated with increased right hemisphere activation in L2. No other study investigated the influence of age of L2 acquisition on language regions in the brain.

Overall, the studies included in the present review used various methods to map different language functions in the brain. Findings were heterogeneous, results varied from identical brain regions to overlapping, but also distinct brain areas for L1 and L2.

## Discussion

To our best knowledge, this study is the first to report interictal language abilities and language mapping in bilingual epilepsy patients based on a systematic review of the literature. Studies differ substantially in patients and controls selection, types of epilepsy, language families tested, number and types of language measures employed, and brain mapping methods applied. Overall, in non-operated epilepsy patients, linguistic abilities in L1 were often better preserved than in L2, but individual results varied from intact language profiles to impairments in several language functions. Results for language mapping varied from identical brain regions for both languages to overlapping, but also distributed cortical areas for L1 and L2.

### Language Abilities in Bilingual Epilepsy Patients

Linguistic abilities in L1 were often better preserved than in L2, however, only few studies investigated the interictal language abilities of their bilingual epilepsy patients.

Naming was the language function most often investigated, and whereas 69% of bilingual epilepsy patients across all studies exhibited intact naming performance in L1, only 12% of them revealed intact naming performance in L2. Two reasons may underly these findings. First, weaker naming abilities in L2 compared to L1 may reflect lower (premorbid) overall language proficiency in L2 compared to L1. Most studies that provided information about language proficiency investigated patients with more than 10 years of exposure to L2 and high proficiency in L2, however, many studies did not present proficiency levels. Furthermore, naming performances in both languages were not controlled for respective proficiency levels, and quantitative information about possible discrepancies between L1 and L2 proficiencies was not given in any study. Second, in chronic epilepsy, neuronal cell loss and deafferentation may affect language associated brain regions, and “weaker” language networks that need to recruit additional neural resources may be more affected than “stronger” networks. In fact, studies on healthy bilinguals have shown that compared to L1, the use of L2 increases activation in language control networks. Explanations for these findings include compensation for lower efficiency in L2, the requirement of more neurons to perform the task (44), and the need to inhibit the “stronger” language in order to access L2 (17).

Besides naming, verbal fluency, reading, writing, and auditory comprehension were investigated, yet just in a small number of patients and with heterogeneous findings. Overall, this systematic review shows that compared to studies in monolingual epilepsy patients, language function in bilingual patients with epilepsy has received far less formal investigation. It therefore underlines the need for a broader range of language assessment and more detailed, standardized information about the proficiency levels in both languages in bilingual epilepsy patients.

### Language Regions in Bilingual Epilepsy Patients

Ten studies were included that investigated language mapping in bilingual epilepsy patients. In bilingual epilepsy patients, the heterogenous picture of language network distributions previously found in bilingual healthy adults was replicated. Whereas, some studies in bilingual patients described overlapping cortical areas for both languages, other studies reported a larger and more bihemispheric distribution for the second language, and again other studies in bilingual epilepsy patients described some shared, but also distinct language areas for both languages.

One study furthermore showed that late age at L2 acquisition was associated with increased right hemispheric involvement (34). This finding is comparable to data in healthy subjects and supports the so-called “critical period hypothesis” which claims that there is an ideal time window to acquire language in a linguistically rich environment, and acquisition of language after that period becomes more effortful and thus needs the recruitment of more brain regions (45). However, the factor age of acquisition is often confounded with the level of proficiency, with earlier age of acquisition being associated with a higher proficiency level. Perani et al. (46) compared two groups of healthy late bilinguals who were either low or high proficient in L2. They found that the proficiency levels were more important than the age of acquisition as determinants of the cortical representation of L2. The influence of both, proficiency levels and age of acquisition seems to vary for different linguistic systems: Wartenburger et al. (47) showed that L2 proficiency predominantly influenced the brain regions involved in semantic decisions in healthy bilinguals, while the age of acquisition of L2 mainly affected the brain regions involved in grammatical processing. In addition to age of acquisition and proficiency, the amount of daily language exposure has also proven to affect the organization of L2 regions in the brain (48). However, none of these language-related factors were investigated in the studies on bilingual epilepsy patients. Moreover, the degree of linguistic relatedness of both languages, i.e., the extend to which first and second languages share semantic, syntactic, and phonological features, may further influence the neural organization in the bilingual's brain. The study sample in this review comprises a wide variety of languages, especially for L1. Whereas some patients spoke two Germanic languages (e.g., Yiddish-English) which share many linguistic features, others spoke two languages which stem from very different language families (e.g., Chinese-English) that have profound differences in their language structures. We hypothesize that the linguistic relatedness of two languages further impacts their neural language organization, though we are not aware of any respective study in healthy bilinguals.

Besides language-related factors, epilepsy-related factors may also influence the organization of two languages in the brain. Cheung et al. (39) showed that left seizure onset lateralization was significantly associated with a more right hemispheric language involvement. No other study included in this review investigated the possible influence of seizure laterality on neural language organization or of other clinical variables. Studies investigating monolingual epilepsy patients demonstrated a significant impact of clinical features inherent in epilepsy that contribute to the neural organization of language in epilepsy, among them seizure frequency, seizure type, age of seizure onset, duration of epilepsy, extent of interictal epileptiform activity, and brain pathology [for review, see Hamberger and Cole (49)]. Thus, it may be hypothesized that these clinical variables add to language-related factors influencing the organization of two languages in the brain and thus add to form the heterogeneous picture found in this review.

Overall, many factors seem to influence the neural language network in bilingual epilepsy patients, and the degree of overlap of two language's brain areas in bilingual individuals planned to undergo epilepsy surgery cannot be predicted to date.

### Limitations

Though broad inclusion criteria, only few studies were identified. Some of them were even more single case studies which did not claim to provide representative data but just presented interesting investigations in single cases. These few studies with an overall low number of participants, however, have used very different methods to map languages in the brain and to examine language abilities in participants with different languages and different proficiency levels. Overall, these factors limit the representativeness of the results of this review and impede to form a consistent picture of neural language organization in bilingual epilepsy patients.

## Conclusions

This review emphasizes the clinical need to individually investigate and map both languages in bilingual epilepsy patients prior to epilepsy surgery. Future research in the field of bilingualism in epilepsy patients should take into account both, language-related and clinical, epilepsy-related variables. Functional brain imaging studies in bilingual epilepsy patients underline the brain's great ability to change and adapt the cortical representation of two languages in the brain.

## Data Availability Statement

Publicly available datasets were analyzed in this study. All data used in this systematic review were published in articles (please see references).

## Author Contributions

LB-D: concept and design, data extraction, analysis and interpretation of data, and drafting of the manuscript. SB: concept and design, data extraction, analysis and interpretation of data, and critical review of the manuscript. All authors approved the submitted version of the article.

### Conflict of Interest

The authors declare that the research was conducted in the absence of any commercial or financial relationships that could be construed as a potential conflict of interest.

## Footnotes

^1^Records meeting more than one exclusion criteria were only counted once.
